# Interrogation of the infarcted and salvaged myocardium using multi-parametric mapping cardiovascular magnetic resonance in reperfused ST-segment elevation myocardial infarction patients

**DOI:** 10.1038/s41598-019-45449-9

**Published:** 2019-06-21

**Authors:** Derek J. Hausenloy, Mei Xing Lim, Mervyn H. H. Chan, Valeria Paradies, Rohin Francis, Tushar Kotecha, Daniel S. Knight, Marianna Fontana, Peter Kellman, James C. Moon, Heerajnarain Bulluck

**Affiliations:** 1 The Hatter Cardiovascular Institute, Institute of Cardiovascular Science, University College London, London, United Kingdom; 2 The National Institute of Health Research University College London Hospitals Biomedical Research Centre, London, United Kingdom; 3 Tecnologico de Monterrey, Centro de Biotecnologia-FEMSA, Nuevo Leon, Mexico; 4 National Amyloidosis Centre, University College London, Royal Free Hospital, London, United Kingdom; 5 National Heart Research Institute Singapore, National Heart Centre Singapore, Singapore, Singapore; 6 National Heart, Lung and Blood Institute, National Institutes of Health, Bethesda, USA; 7 Cardiovascular and Metabolic Disorders Program, Duke-National University of Singapore, Singapore, Singapore; 8 Yong Loo Lin School of Medicine, National University Singapore, Singapore, Singapore; 9 Golden Jubilee National Hospital, Clydebank, Glasgow, United Kingdom

**Keywords:** Cardiology, Interventional cardiology

## Abstract

We used multi-parametric cardiovascular magnetic resonance (CMR) mapping to interrogate the myocardium following ST-segment elevation myocardial infarction (STEMI). Forty-eight STEMI patients underwent CMR at 4 ± 2 days. One matching short-axis slice of native T1 map, T2 map, late gadolinium enhancement (LGE), and automated extracellular volume fraction (ECV) maps per patient were analyzed. Manual regions-of-interest were drawn within the infarcted, the salvaged and the remote myocardium. A subgroup analysis was performed in those without MVO and with ≤75% transmural extent of infarct. For the whole cohort, T1, T2 and ECV in both the infarcted and the salvaged myocardium were significantly higher than in the remote myocardium. T1 and T2 could not differentiate between the salvaged and the infarcted myocardium, but ECV was significantly higher in the latter. In the subgroup analysis of 15 patients, similar findings were observed for T1 and T2. However, there was only a trend towards ECV_salvage_ being higher than ECV_remote_. In the clinical setting, current native T1 and T2 methods with the specific voxel sizes at 1.5 T could not differentiate between the infarcted and salvaged myocardium, whereas ECV could differentiate between the two. ECV was also higher in the salvaged myocardium when compared to the remote myocardium.

## Introduction

Myocardial salvage index (MSI) is considered a more sensitive measure for assessing the efficacy of a cardioprotective strategy than an absolute reduction in myocardial infarct (MI) size, as it normalizes the reduction in MI size to the area-at-risk (AAR)^1–3^. The AAR refers to the territory supplied by the infarct-related artery that would have been irreversibly injured without reperfusion. Once the AAR and MI size are known, MSI can be calculated using the formula: myocardial salvage index = (AAR − MI size)/AAR^4^.

Cardiovascular magnetic resonance imaging (CMR) is considered the reference standard technique for MI size quantification^5–7^. It can also provide information on the edema-based AAR using T2-weighted imaging^3,8,9^, T2 mapping^10,11^, T1 mapping^11,12^, early gadolinium enhancement^13^, or post-contrast steady-state-free-precession (SSFP) cine images^14^. However, none of these techniques have been established as the reference standard in the clinical setting for the AAR by CMR. Controversies exist on whether edema is confined to the infarcted myocardium only^15^, or extends to the salvaged myocardium^16^.

Conventionally, MI size is quantified on the late gadolinium enhancement (LGE) imaging following contrast administration^4^. Native T1 and T2 mappings are acquired prior to contrast administration. A recent pre-clinical study^16^ using a canine model of reperfused MI has reported that native T2 values were higher in infarcted myocardium when compared to salvaged myocardium. However, the same group reported no difference in native T1 values between the infarcted and salvaged myocardium, but post-contrast T1 was able to differentiate between the infarcted and salvaged myocardium, 8 minutes following contrast injection^17^. So far, no clinical studies have interrogated the infarcted and salvaged myocardium in the same patients using the latest T1, T2 and ECV mapping technology. If we were able to differentiate between the infarcted and salvaged myocardium using native T1 and T2 mapping, we could potential quantify both the AAR and MI size accurately without contrast on these maps by applying different thresholds, which would significantly shorten scan time for these patients and expand the availability to those with contraindications to the gadolinium chelate.

In order to clarify whether the infarcted myocardium can be distinguished from the salvaged myocardium in the acute setting, we used multiparametric CMR with native T1 maps, T2 maps and automated extracellular volume fraction (ECV) mapping to interrogate the infarcted and salvaged myocardium in patients shortly after ST-segment elevation myocardial infarction (STEMI) reperfused by primary percutaneous coronary intervention (PPCI).

## Materials and Methods

### Study population

Patients included in this study have been previously reported^18–25^ but no prior publications have addressed multiparametric mapping in the salvaged and the infarcted myocardium. In brief, the London-Harrow Research Ethics Committee approved this study. Fifty STEMI patients were prospectively recruited from August 2013 to July 2014 following informed consent. This study complied with the declaration of Helsinki and was approved by the local ethics committee. Forty-eight patients completed the first CMR at 4 ± 2 days post-PPCI. A subgroup analysis was performed in those patients without late microvascular obstruction (MVO) (as this would interfere with the T1 and T2 of the infarcted myocardium) and with ≤75% transmural extent of infarct (to minimize partial volume effects when drawing the regions of interest (ROI) in the salvaged myocardium).

### Cardiac MRI acquisition

All CMR scans were performed on a 1.5 Tesla scanner (Magnetom Avanto, Siemens Medical Solutions) using a 32-channel phased-array cardiac coil. The imaging protocol included short axis coverage of cines, native Modified Look-Locker Inversion Recovery (MOLLI) T1 mapping, T2 mapping, LGE and post-contrast T1 mapping. All the short axis images were aligned with the cine short axis slice position.

### Native T1 mapping (Work in progress 448B, siemens healthcare)

An SSFP-based MOLLI sequence with a sampling protocol of 5 s(3 s)3 s was used for the T1 maps. The acquisition parameters were: flip angle = 35°; pixel bandwidth 977 Hz/pixel; matrix = 256 × 144; echo time = 1.1 ms; voxel size = 1.5 × 2.0 × 6.0 mm. Motion correction and a non-linear least-square curve fitting of the set of images acquired at different inversion times were performed inline by the scanner to generate a pixel-wise colored T1 map^26^.

### T2 Mapping (Work in progress 448B, siemens healthcare)

Pixel-wise colored T2 maps were generated inline following motion correction and fitting to estimate T2 relaxation times^27^ after acquiring 3 single-shot images at different T2 preparation times (0 ms, 24 ms, and 55 ms, respectively) and the acquisition parameters were: flip angle = 70°; pixel bandwidth 930 Hz/pixel; matrix = 116 × 192; echo time = 1.1 ms; repetition time = 3xR-R interval; voxel size = 2.0 × 2.7 × 6.0 mm.

### Late gadolinium enhancement

LGE images were acquired 10 to 15 minutes after the injection of 0.1 mmol/kg of Gadoterate meglumine (Gd-DOTA marketed as Dotarem, Guerbet S.A., Paris, France), using either a standard segmented ‘fast low-angle shot’ two-dimensional inversion-recovery gradient echo sequence LGE phase-sensitive inversion recovery (PSIR) sequence or a respiratory motion-corrected, free-breathing single-shot SSFP averaged PSIR sequence^28^.

### Post-contrast T1 mapping (Work in progress 448B, siemens healthcare)

Post-contrast T1 maps were obtained using the 4 s(1 s)3 s(1 s)2 s sampling protocol (to improve the accuracy of T1s in the 200–600 ms range as previously described^29^) 15 minutes after contrast injection (0.1 mmol/kg of Dotarem) using similar acquisition parameters (voxel size = 1.5 × 2.0 × 6.0 mm) as for native T1 maps.

### ECV mapping

The previously described and validated automated method for producing a pixel-wise ECV map was used^30^ with motion-correction and co-registration of the native and post-contrast T1 pixel maps. Each patient had hematocrit checked at the time of the scan and a pixel by pixel, re-motion corrected and re-registered map was created (ECV Mapping Tool, Version 1.1)^30^ using the following formula^31^:

$$ECV=(1-haematocrit)\times \frac{(1/T1myocardium\,post)-(1/T1myocardium\,pre)}{(1/T1blood\,post)-(1/T1blood\,pre)}$$

### Imaging analysis

All imaging analysis was performed using CVI42 software (V.5.1.2 (303), Calgary, Canada) as previously described^18,25^. In brief, MI size (from LGE images) and AAR (from T2 maps) were quantified from the whole LV coverage short axis acquisitions using signal intensity thresholds of 5 and 2 standard deviations (SD) above the normal remote myocardium^32^, respectively, and expressed both as a percentage of the left ventricle (%LV) and in grams. Transmural extent of infarct (TEI) was quantified by averaging the values from 100 chords from each short axis slice to obtain the mean transmural extent of LGE for each of the 16 American Heart Association (AHA) segments and expressed in as a continuous variable and then objectively classified into groups of “no LGE”, “1–25%TEI”, “26–50%TEI”, “51–75% TEI” and “76–100%TEI^25^”.

**Figure 1:**
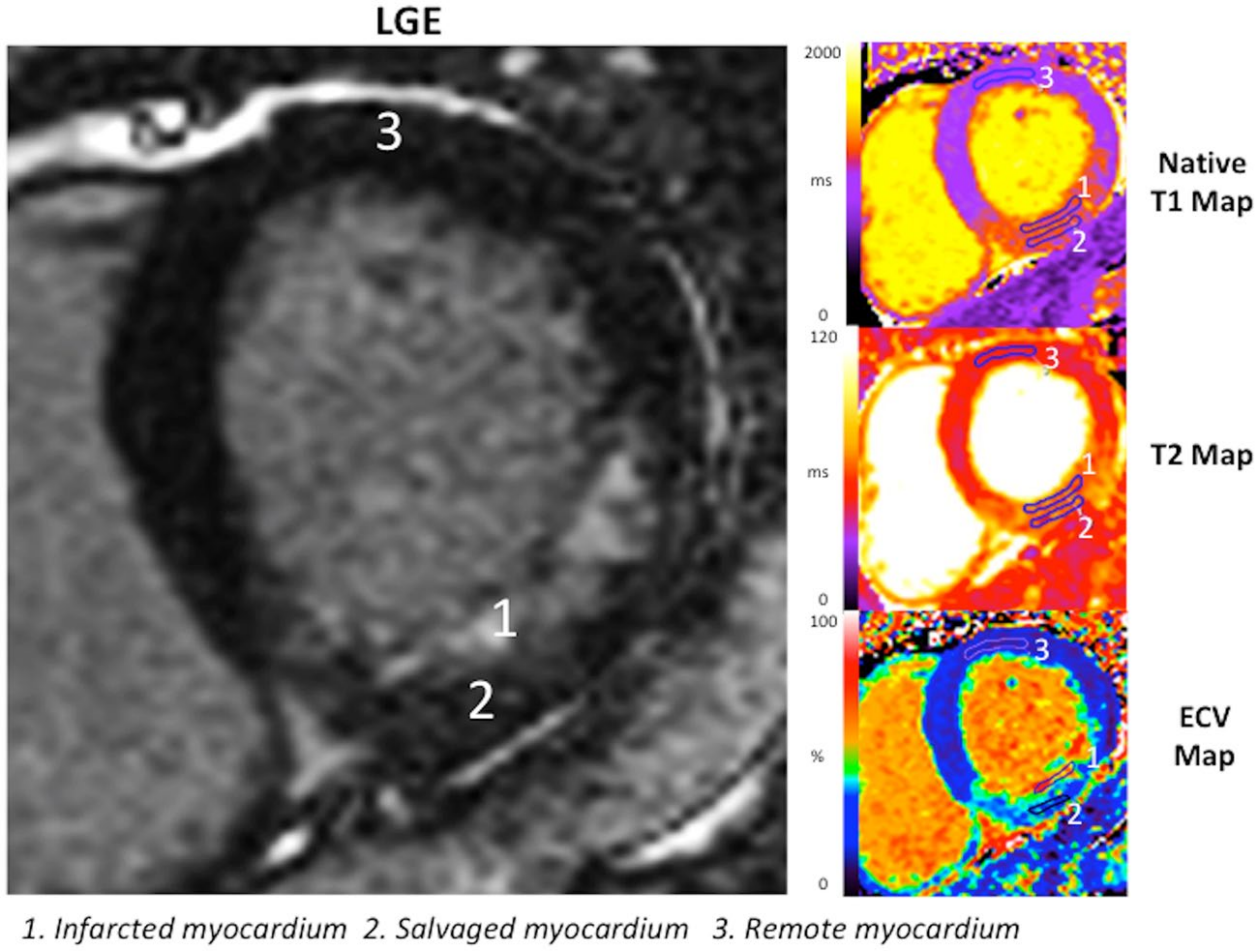
Example of matching maps and LGE image per patient analyzed for this study. This is an example of the native T1, T2 and ECV maps for a patient with an acute inferior STEMI. Using the LGE image as reference, manual ROIs were drawn in infarcted (No. 1), salvaged (No. 2) and remote myocardium (No. 3) on the native T1, T2 and ECV maps.

For representative parametric data in the infarcted, salvaged and remote myocardium for each patient, one short-axis slice per patient of matching native T1 map, T2 map, ECV map through the region of wall motion abnormality was analyzed and the corresponding LGE image was used as the reference. ROIs were manually delineated in the infarcted, salvaged and the remote myocardium as shown in Fig. 1. ROI delineation in the infarcted and salvaged myocardium were performed using a similar approach to a previous study (infarcted myocardium identified as the region of hyperenhancement on LGE images while salvaged myocardium identified as the non-enhancing myocardium epicardial to the infarcted myocardium)^16^. After identifying the infarcted and salvaged myocardium on LGE, ROIs were placed in the corresponding regions on the native T1 maps and copied to the T2 and ECV maps, with minimal manual adjustments when required to avoid partial volume effects. The remote myocardium was defined as the region that did not display any LGE, native T1 or T2 abnormalities in a segment that was 180° from the infarct-related territory. In those with >75%TEI, no ROI were available for the salvaged myocardium. Patients without MVO (to avoid the interference of the hypointense core on the T1 and T2 values of the infarcted myocardium) and with ≤75%TEI (to minimize partial volume effects when drawing ROI in the salvaged myocardium) were included in the subgroup analysis. In order to evaluate whether partial volume effect may impact on our results, we also excluded those patients with TEI 51–75% in a further subgroup analysis.

### Statistical analysis

Statistical analysis was performed using SPSS version 22 (IBM Corporation, IL). Normality was assessed using Shapiro-Wilk test. Continuous data were expressed as mean ± SD or median (interquartile range), and compared with the paired Student t-test/Wilcoxon signed rank test or unpaired Student t test/Mann–Whitney U test, where appropriate. Categorical data were reported as frequencies and percentages. To compare T1, T2 and ECV values in the remote, salvaged and infarcted myocardium, a linear mixed model was used with the T1, T2 or ECV being the dependent variable; the patients being a covariate; and the territory within patients (remote, salvaged, infarcted) being a fixed factor, to take into account interaction among segments within the same patients. Pairwise comparison was performed using Bonferroni correction. All statistical tests were 2-tailed, and P < 0.05 was considered statistically significant.

## Results

Baseline characteristics of the patients are shown in Table 1. CMR was performed at 4 ± 2 days. The median age of the patients was 60 (49–69) years old and the majority of patients (40/48, 83%) were male. The infarct-related coronary artery distribution was as follows: left anterior descending artery in 28/48 (58%) of patients; right coronary artery in 18/48 (38%); and circumflex artery in 2/48 (4%), (Table 1).

**Table 1:** Patients’ baseline characteristics.

Details	Number	
	N = 48 (whole cohort)	N = 15 (subgroup)
Number of patients	48	15
Male (%)	40 (83)	13 (87)
Age (year)	60 [49–69]	56 [42–67]
Diabetes Mellitus (%)	9 (19)	5 (33)
Hypertension (%)	15 (31)	3 (20)
Smoker (%)	15 (31)	6 (40)
Dyslipidemia (%)	15 (31)	5 (33)
LAD (%)	28 (58)	5 (33)
Cx (%)	2 (4)	1 (7)
RCA (%)	18 (38)	9 (60)
Onset-to-balloon time (mins)	182 [128–328]	178 [129–326]
MI size (%LV)	26 [18–38]	19 [10–28]
AAR (%LV)	43 [33–52]	39 [31–53]
MVO (%)	30 (63)	0
LV End-diastolic volume (ml)	162 [138–195]	152 [132–170]
LV End-systolic volume (ml)	81 [62–112]	67 [62–93]
LV Ejection fraction (%)	49 [43–58]	52 [46–59]
LV Mass (g)	113 [92–132]	120 [81–133]

The interquartile ranges for the medians are enclosed using the square brackets. LAD: left anterior descending artery; Cx: circumflex artery; RCA: right coronary artery; MI: myocardial infarct; AAR: area-at-risk; LV: left ventricle.

403/768 (53%) myocardial segments had no LGE. Out of those segments with LGE, the TEI were as follows: 1–25%TEI: 89/365 (24%); 26–50%TEI: 100/365 (27%); 51–75% TEI: 90/365 (25%); and 76–100%TEI: 86/365 (24%). 15 patients without MVO and with ≤75%TEI were included in the subgroup analysis (Table 1).

### Whole cohort (n = 48)

**Figure 2:**
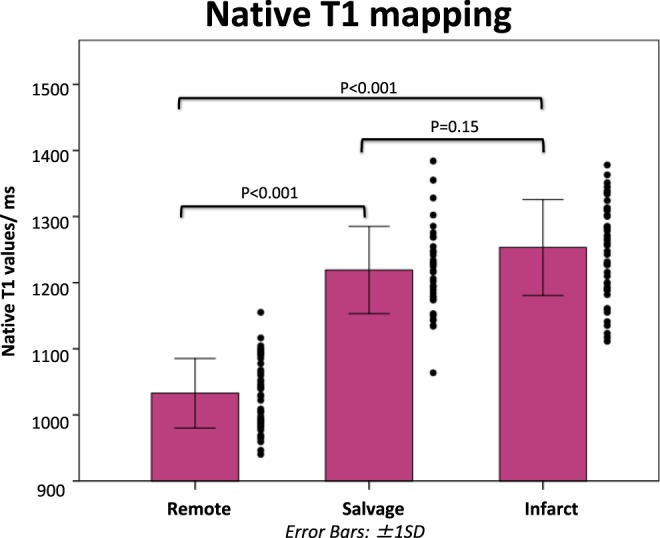
Comparison of native T1 values in the infarcted, salvaged and remote myocardium. This bar chart compares the native T1 values in the infarcted, salvaged and remote myocardium. Native T1 could not differentiate between the salvaged and the infarcted myocardium.

T1_infarct_ (1253 ± 73 ms) and T1_salvage_ (1219 ± 66 ms) were both higher than T1_remote_ (1033 ± 53 ms, P < 0.001 for both) with no significant difference between T1_infarct_ and T1_salvage_ (P = 0.15) (Fig. 2).

**Figure 3:**
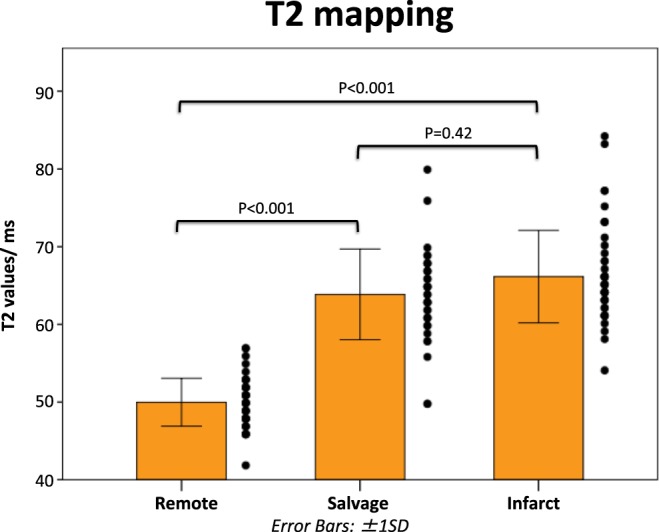
Comparison of T2 values in the infarcted, salvaged and remote myocardium. This bar chart compares the T2 values in the infarcted, salvaged and remote myocardium. T2 could not differentiate between the salvaged and the infarcted myocardium.

T2_infarct_ (66 ± 6 ms) and T2_salvage_ (64 ± 6 ms) were significantly higher than T2_remote_ (50 ± 3 ms, P < 0.001 for both comparison) with no significant difference between T2_infarct_ and T2_salvage_ (P = 0.42) (Fig. 3).

**Figure 4:**
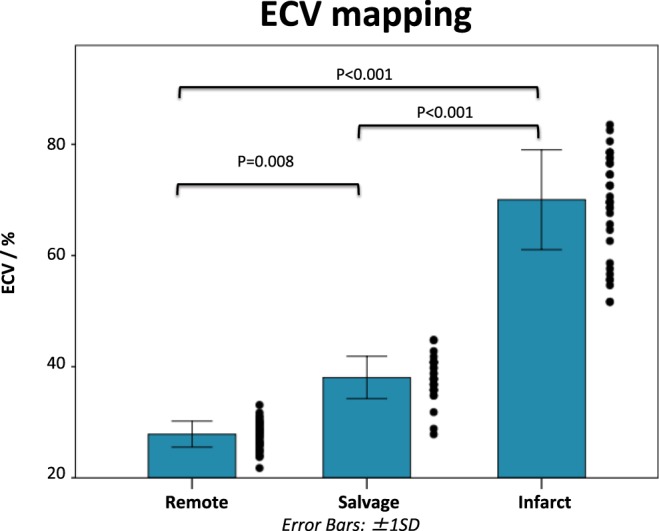
Comparison of ECV values in the infarcted, salvaged and remote myocardium. This bar chart compares the ECV values in the infarcted, salvaged and remote myocardium. ECV could differentiate between the salvaged and the infarcted myocardium.

ECV_infarct_ (70 ± 9%) was significantly higher than ECV_salvage_ (38 ± 4%) and ECV_remote_ (28 ± 2%, P < 0.001 for both comparisons). Furthermore, ECV_salvage_ was also significantly higher than ECV_remote_ (P < 0.001) (Fig. 4).

### Subgroup analysis (n = 15)

A similar pattern was observed for T1 and T2 in the subgroup of patients without MVO and with ≤75%TEI. T1_infarct_ (1283 ± 81 ms) and T1_salvage_ (1235 ± 61 ms) were significantly higher than T1_remote_ (993 ± 49 ms, P < 0.001) with no significant difference between T1_infarct_ and T1_salvage_ (P = 0.38).

T2_infarct_ (64 ± 5 ms) and T2_salvage_ (62 ± 7 ms) were significantly higher than T2_remote_ (48 ± 3 ms, P < 0.001) and there was no significant difference between T2_infarct_ and T2_salvage_ (P = 0.45).

As for ECV, ECV_infarct_ (60 ± 10%) was significantly higher than ECV_salvage_ (37 ± 5%, P < 0.001) and ECV_remote_ (24 ± 3%, P < 0.001). However, there was only a trend towards ECV_salvage_ being higher than ECV_remote_ but this comparison did not reach statistical significance (P = 0.07).

In order to evaluate whether partial volume effect may have contributed to some extent to the above results, we further excluded those patients with TEI 51–75% (n = 3). The same findings were seen for T1 (T1_infarct_; 1293 ± 86 ms; T1_salvage_: 1243 ± 63 ms; T1_remote_: 993 ± 54 ms), T2 (T2_infarct_; 64 ± 5 ms; T2_salvage_: 63 ± 7 ms; T2_remote_: 48 ± 3 ms) and ECV (ECV_infarct_; 61 ± 11%; ECV_salvage_: 36 ± 5%; ECV_remote_: 24 ± 3%). There was no significant difference between T1_infarct_ and T1_salvage_ (P = 0.27) and between T2_infarct_ and T2_salvage_ (P = 0.99) but there was a significant difference between ECV_infarct_ and ECV_salvage_ (P = 0.001).

## Discussion

This study shows that both infarcted and salvaged myocardium have elevated T1 and T2 but whilst the ECV was very elevated in infarction, it was partly elevated in salvage, with implications for salvage detection (T1 and T2 not useful alone), and for our understanding of salvage.

Although the standard deviation of the T1 in the remote myocardium appeared relatively high (±53 ms), we have previously reported reference values by MOLLI in a healthy control group to be 1000 ± 25 ms^19^. Therefore, the larger variability in T1 in the remote myocardium in this cohort is unlikely due to the precision of the T1 mapping technique used, but rather changes in the remote myocardium that us^19^ and other groups^33,34^ have reported upon previously.

Our results are consistent with the findings of previous studies showing that the increase in native T1 and T2 values in the infarct-related artery corresponded to the edema-based AAR in the clinical setting^10–12,35^. The potential explanation for our observations could be related to the difference in severity of the intracellular and extracellular edema in the infarcted and salvaged myocardium. Native T1 and T2 measure edema both in the intracellular and extracellular compartments, whereas ECV measures edema in the extracellular compartment only. Native T1 and T2 may be similarly affected by intracellular edema, and may not be able to differentiate the severity of extracellular edema in some patients. Following contrast administration, the kinetics of the gadolinium chelate wash-in and wash-out at pseudo-equilibrium would be different in the infarcted (likely to predominantly consist of cardiomyocytes with ruptured cell membranes and extracellular edema) and salvaged myocardium (likely to predominantly consist of cardiomyocytes with intact cell membrane and with both intracellular and extracellular edema). A larger concentration of contrast would be present in the infarcted myocardium than in the salvaged myocardium (guided by the severity of extracellular edema) and this would explain the difference in the findings between pre-contrast and post-contrast mapping parameters. It is not surprising that ECV was significantly different between the infarcted and salvaged myocardium and this likely reflects the different severity of extracellular edema in those two territories. In those with significant myocardial salvage, the extracellular edema may be less severe as shown by the ECV values in our subgroup analysis where there was some overlap between the salvaged and remote myocardium.

However, Hammer-Hansen *et al*.^16^ recently reported in a canine model of reperfused MI that T2 values in the infarcted myocardium were higher than in the salvaged myocardium, and both were significantly higher than the remote myocardium. They only included 11 animals, and they all underwent mid left anterior descending artery ligation for 2 hours, and CMR was performed at similar times for all animals. The difference between the pre-clinical and clinical findings may be related to the animal species used (canines being more prone to develop collaterals) or the heterogeneity of the patient selection (patients presenting with a wide duration of symptoms, with STEMI in all territories and with CMR performed at different time points post-PPCI). Furthermore, the occlusion of the infarct-related artery was abrupt in the animal model whether was this process is slower and stuttering in the clinical setting with downstream embolism of plaque debris and microemboli. Although we also performed a subgroup analysis by excluding patients with MVO (to avoid the interference of the hypointense core on the T1 and T2 values of the infarcted myocardium) and >75% TEI (to minimize partial volume effects when drawing ROI in the salvaged myocardium), some patients may still have had minute areas of MVO and haemorhage within the infarcted myocardium that were not visible to the naked eye and may have affected the native T1 and T2 values within the infarcted myocardium.

In the clinical setting, Hammer-Hansen *et al*.^17^ also showed that native T1 was similar in the infarcted and salvaged myocardium, and our findings confirm this. If T1 and T2 mapping could differentiate between infarcted and salvaged myocardium at 1.5 T in the acute setting, then by applying a particular threshold, it would possible to delineate the acute MI size without contrast. Interestingly, Liu *et al*.^36^ recently showed that native T1-mapping could identify acute MI size. However, their study was performed on a 3 T scanner and only 58 short-axis T1 maps without microvascular obstruction were analyzed in that study^36^. Further work is required with a larger number of patients to evaluate whether native T1 mapping can delineate the acute MI size.

ECV mapping at pseudo-equilibrium provides pixelwise quantification of the ratio of the extracellular compartment to the total myocardial volume. Hwang *et al*.^37^ previously showed that a threshold of ECV >0.42 or 0.44 could be used to quantify the extent of scars in the setting of chronic MI and hypertrophic cardiomyopathy. Our findings are consistent with those from Hammer-Hansen *et al*.^17^, who showed that the ECV of the infarcted myocardium was higher than that of the salvaged myocardium. They also showed that the ECV of the salvaged myocardium was higher than that of the remote myocardium. Most recently, Garg *et al*.^38^ showed that ECV maps could be used for the AAR and chronic MI size by applying different thresholds. However, the limits of agreement with the T2-derived AAR from the T2-weighted imaging were quite wide at ±10.4%^39^. Whether the wide limits of agreement in edema measures seen in the study by Garg *et al*.^39^ could be due to ECV not identifying the edema-based AAR accurately in some patients with less severe extracellular edema, warrants further investigation.

Partial volume effects may have affected these results. Further analysis of those with TEI up to only 50% resulted in finding consistent with our prior findings. However, this does not necessarily suggest that partial volume effects along the slice direction are not important. Despite this, this highlights the fact that in the clinical setting, using current T1 and T2 mapping with the current voxel sizes at 1.5 T, salvaged myocardium cannot be differentiated from the infarcted myocardium.

### Limitations

The number of patients included in our study was small and therefore any further in-depth analysis of any temporal trend, relationship with other predictors and indicators of myocardial injury (symptom duration, patency of infarct-related artery and cardiac enzymes) were not performed. However, a retrospective sample size calculation to detect a 25% difference between the infarcted and remote myocardium showed that the study was adequately powered. CMR was performed at different days within the first week post-STEMI and may be a source of bias, given that edema has been recently shown to be dynamic^40^. Our delineation of the infarcted and salvaged myocardium was based on the concept of ‘wave-front phenomenon’ where the ischemic injury is thought to progress radially from the subendocardium to the epicardium with increasing duration of coronary occlusion^41^, and therefore the epicardial side of the infarcted myocardium was considered as salvaged myocardium. This is the same definition used by Hammer-Hansen *et al*.^16^. We only analyzed one short axis slice of T1, T2 and ECV maps per patient although this is similar to previous studies^16,17^. The spatial resolution of T1, T2, and ECV maps were not the same.

## Conclusion

In this cohort of STEMI patients, current native T1 and T2 methods with the specific voxel sizes at 1.5 T could not differentiate between the infarcted and salvaged myocardium, whereas ECV could differentiate between the two. ECV was also higher in the salvaged myocardium when compared to the remote myocardium. Whether this is due to a true pathophysiological observation or partial volume effect warrants further investigation.
